# Production of gathering spaces in post-disaster recovery scenarios: case studies from the Great East Japan Earthquake and Tsunami-2011

**DOI:** 10.1186/s40410-023-00195-4

**Published:** 2023-03-23

**Authors:** Yegane Ghezelloo, Akihiko Hokugo, Osamu Tsukihashi

**Affiliations:** 1 Graduate School of Engineering, Department of Architecture, Kobe University, Rokkodai, Nada, Kobe, Hyogo 657-8501 Japan; 2 Graduate School of International Cooperation Studies, Graduate School of Engineering, Research Center for Urban Safety and Security, Kobe University, Rokkodai, Nada, Kobe, Hyogo 657-8501 Japan

**Keywords:** Gathering space, Hiroba, Community-driven recovery, Government-led recovery, Production of space

## Abstract

Gathering and public spaces, along with infrastructure and houses, are demolished because of disasters, which weakens the community ties. Different approaches, such as government-led and community-driven, to recovery initiate the recovery of gatherings and public spaces, and the long-term impact of each recovery approach on community recovery may not be overseen. This study attempts to determine incorporation of community participation in different recovery approaches and its corresponding result in the production of gathering spaces, based on two main background theories: Arnstein’s ladder of citizen participation and Henri Lefebvre’s production of space triad. We attempted to determine the results by reviewing case studies with different recovery processes after the Great East Japan Earthquake and Tsunami-2011 and through interviews and questionnaire surveys. The results showed that the production of gathering spaces may be associated with the recovery scenario in each case study. In community-driven cases, the main gathering spaces are small open spaces, evenly superimposed and accessible, and diverse in spatial configuration, provide services for the users at a good level, and are in a sync with other gathering spaces. By contrast, in government-led cases, gathering spaces contain primary and secondary spaces that lack connections with each other. These main gathering spaces are centralized near disaster public housing sites, are highly accessible to disaster public housing residents, provide a high range of leisure-based activities, and provide services to users from inside and outside of the communities. These main gathering spaces are extended by inclusive open space (Hiroba) and this spatial planning is closer to the concept of public spaces compared to others.

## Introduction

Many scholars in the disaster recovery field are concerned with recovery approaches, levels of residents’ participation, public and gathering space recovery, and their benefits for disaster-affected communities. This study attempts to address these concerns and reviews the levels of embodiment of community participation and gathering space production in different recovery approaches, led by government and communities, in the post-Great East Japan Earthquake and Tsunami 2011 (GEJET-2011) recovery processes. This research considers two main background theories: Arnstein’s ladder of citizen participation and Henri Lefebvre’s production of the space triad in selected case studies from the Tohoku region of Japan and investigates the hypothesis based on field visits, interviews, and questionnaire surveys. Arnstein’s ladder of citizen participation has inspired and supported numerous studies in the fields of sociology and space design, from the normal timeline of a community to disaster-affected periods. However, Henri Lefebvre's production of the space triad emphasizes the experiences of space production, and differences in the spatial practices and users’ experiences are the outcomes of the initial planning and decision making. This concept is mainly focused on during postmodern acts of urban planning and requires a more in-depth study of disaster recovery processes.

### Literature review

To determine the production process of gathering spaces in case studies with different recovery scenarios (government-led and community-driven) after GEJET-2011, we reviewed the background theory and similar studies; the literature review part tries to draw the concepts of the hypothesis. First, gathering spaces, public spaces, and their differences in Japanese society is defined. Second, Lefebvre’s triad of space production is introduced. Finally, the concept of recovery and the importance of participatory recovery by emphasizing Arnstein’s ladder of citizen participation in community recovery are discussed and represented in gathering spaces.

#### Gathering spaces

Gathering spaces are spaces that are physically and intellectually open to everyone and not advantageous to anyone. What is referred to in this research as gathering spaces fits Habermas’s definition of public spaces but in the boundaries of the built space which is “Platforms/spaces/places that are accessible to everyone, no one enters them with an advantage over another, and they have a potential foundation for a critique of society based on democratic principles (Holub 1991).” However, in the Japanese context, the concept of globally defined public spaces is not widely understood, and local understanding and implemented environments of such places are different from global common knowledge. Considering this, the term “gathering space” is used instead of “public space” to avoid misinterpretation as referring only to government-owned/organized places (Dimmer 2012) in this research.

For the purposes of this research, explaining the reason of gathering spaces being among the most important types of produced and recovered places is essential. Haas and others (Haas et al. 1997) described gathering spaces as environments that emerge since before disaster periods into recovery periods as makeshift sociophysical places. These places help affected residents function as communities by providing a platform for individuals and groups to interact. Klinenberg (2018) conducted a study on post disaster social capital and communities and identified shared spaces and community gathering places as key to recovering communities and reaching democratic societies with shared values.

Nelan and Schumann (2018) also investigated post-disaster gathering space recovery in aftermath of Hurricane Harvey and identified following three dimensions of place attachment (Low and Altman 1992) in connection with gathering spaces: the bonding of people to certain places, symbolic and emotional meaning through routine interpersonal exchanges, and place dependence based on the multiple functions of the space. Nelan and Schumann also identified displacement of individuals, lack of formal emotional support centers, and lack of communal recovery as the main issues that gathering space recovery could address (Nelan and Schumann Iii 2018).

As mentioned, the definition of public spaces in Japan is different from the global understanding. Global concept of open spaces, public gathering spaces, and public facilities might also be considered public spaces (such as parks, plazas, and libraries) and other public spheres (street, roads, and pedestrian paths). In Japanese urban planning, spaces and activities implemented by the government sector are called “public-public,” such as schools and gymnasiums, while other public spaces and activities handled by the private sector are referred to as “private–public,” such as community centers and meeting places (Aota 2012; Dimmer 2012). In Japan, authorities are in charge of managing the open spaces or Hiroba (wide-open area, referring to the physical condition rather than any social or formal property of the actual space) and primarily maintain these spaces for post-disaster evacuation and other emergencies. On the other hand, some gathering spaces that are considered social spaces for residents’ participation and gatherings and are usually within closed boundaries and territorial buildings and sometimes consist of exclusive open spaces added to closed spaces (Aota 2012; Kuma et al. 2015; Okabe 2017), such as public halls, community centers, and meeting places. In this research, Hiroba (wide-open area) is considered to be totally accessible, inclusive, and open space inclusively available to everyone and without means of obstacles of use, such as usage fees, guardrails, walls, or fences, and curfew that limit user access. However, open spaces belonging to buildings that limit the access of users by fences, walls, or guardrails and charges for use are called exclusive open spaces.

#### Production of gathering spaces

**Fig. 1:**
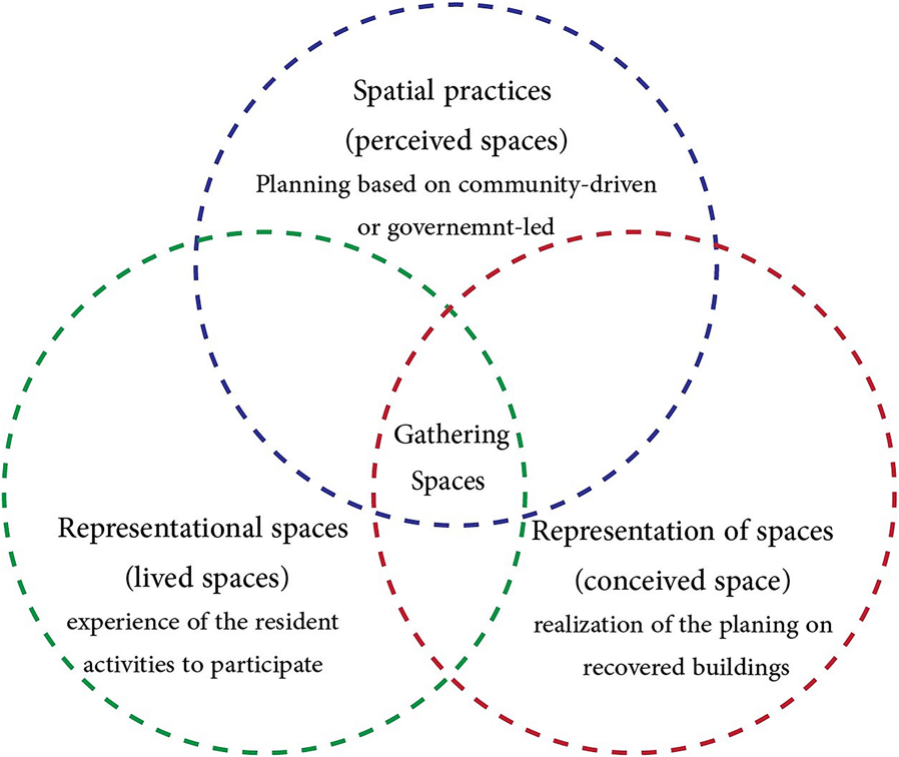
Triad of space production, revised by authors

Lefebvre defined space and social spaces as a product of stakeholders’ decisions and user experiences based on a triad model. This model provides a framework for recognizing the three elements of production of space (Fuchs 2019). Lefebvre’s production of space refers to the spatial triad framework and consists of (a) spatial practices (perceived space), (b) representations of space (conceived space), and (c) representational space (lived space) (Gregory 1995). In fact, space production is based on relationships between planning attempts (triad 1), physical configurations and design (triad 2), and individuals’ experiences in the produced spaces (triad 3). This relationship emphasizes the theory of the dimensions of place attachment, especially on representational spaces (Low and Altman 1992). Figure 1 illustrates the triad of social spaces in the gathering spaces.

#### Residents’ participation

Even though national and local governments provide the main logistics, budget, and knowledge of recovery, they work as urban interventions in the environment of disaster-stricken communities as a series of long- and short-term planning (Lee et al. 2022). However, unforgettably, residents are an important part of the environment on which recovery plans are based, and they should be engaged in both long- and short-term recovery planning processes. Although scholars have underlined different aspects of recovery and emphasized the importance of communities, planners and stakeholders generally focus on infrastructure, housing reconstruction, and economic growth. Many authors have presented the risks of perspectives of government leadership that do not meet community needs and maintain distance from people and communication between communities and stakeholders (Aldrich 2017; Dimmer 2012; Murakami et al. 2014). To achieve better city recovery for residents, bridging this gap between a demonstration pilot project and people as an equitable approach to disaster reconstruction at a scale that can benefit all survivors is essential (Maly 2018). Numerous definitions of community driven and participatory governance approaches have been issued by governments and stakeholders. The Australian Institute of Family Studies (Moore et al. 2016) describes participation and community-based activities as community engagement, which is a key strategy for improving residents’ outcomes.

The field of participatory governance has been well identified and debated by several contributors, and definitions have been established for this topic, such as co-design and co-production. Public participation levels can vary widely, starting from informing the locals, consulting with them, involving them, collaborating with them, and empowering them. Arnstein’s ladder of participation and her definitions of levels of involvement in the decision-making process range from the lowest level (manipulation–non-participation) to the highest (citizen control–citizen power). She also described citizen power as comprising partnership, delegated power, and citizen control levels; in this way, citizen power can be achieved in the decision-making process (Arnstein 1969, 2019; Fung 2015; IAP2 2022). Co-production and co-design of space can be an extension of residents' participation in planning and space design as relational processes that involve a range of different actors—government bodies, residents associations, NPOs, and individuals—with diverse capacities to enhance outcomes on the ground, interact with each other, and recalibrate multiple relations with governance to produce shared spaces in diverse ways, resulting in more inclusive public spaces (Gururani and Kennedy 2021; Murphy et al. 2022; Roy and Ong 2011). However, to achieve the best outcomes, this process requires step-by-step practice and planning by the collected working groups representing the needs and demands of the majority and minority members of the community (O’Reilly et al. 2022; Reed et al. 2022).

**Fig. 2:**
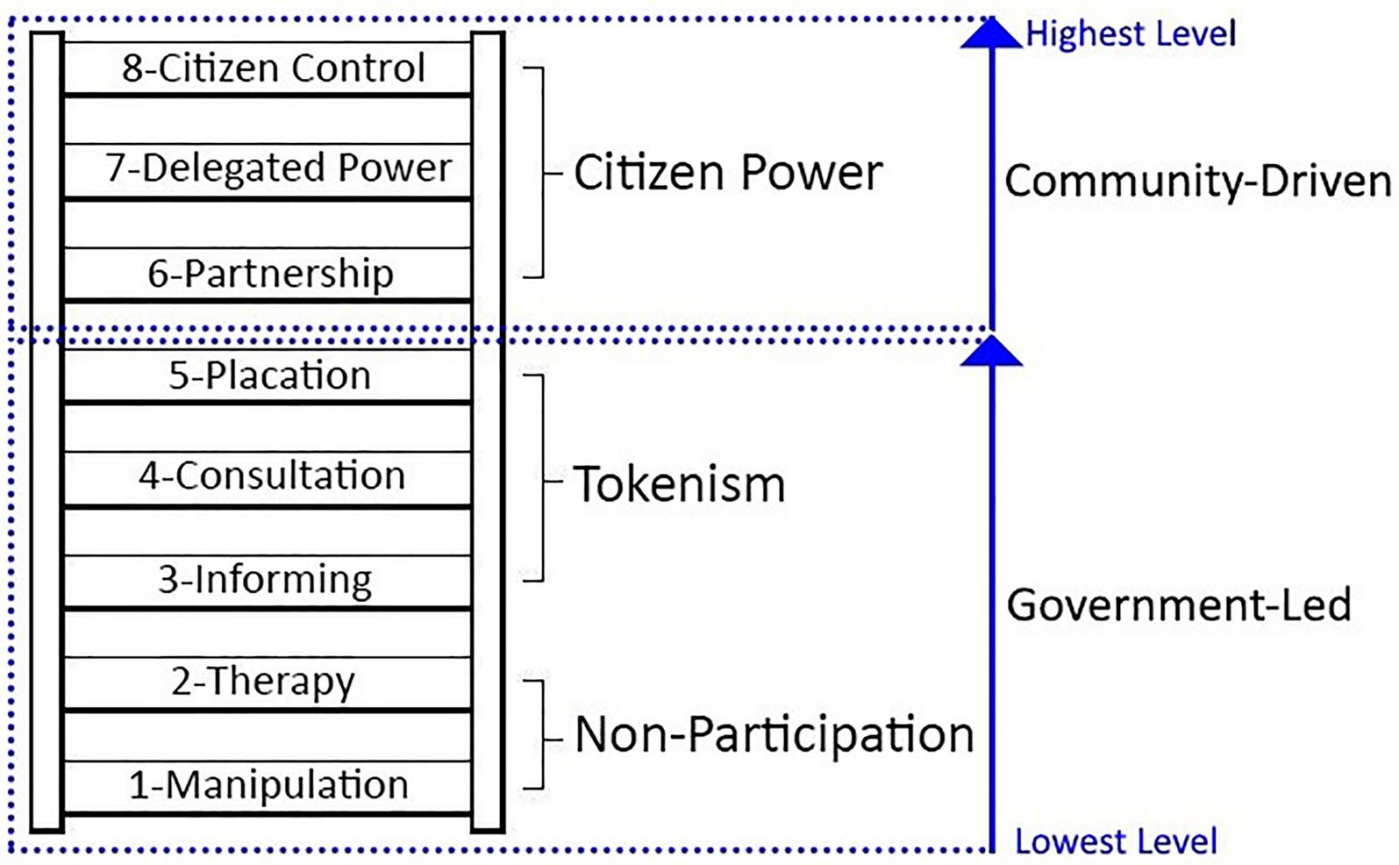
Ladder of citizen participation by Arnstein, revised based on authors’ observations

Figure 2 shows Arnstein’s ladder of citizen participation in combination with the recovery scenarios expressed in this study. Government-led and community-driven recovery scenarios were derived from the results of observations of case studies by the authors.

##### Japanese context

In Japan, with its long history of disaster and reconstruction experience, aside from government-led processes for recovering housing, infrastructure, industries, and transportation, residents have their own share of decision making and partnerships in the process. It is also highly recommended that, even while living in temporary housing and recovered areas, consideration be paid to the recovery of gathering spaces as one of the most important places to make community recovery tangible. Community-driven town planning and decision making in Japan is called machizukuri, which is also used to refer to recovery processes; however, the implementation level of machizukuri differs from one municipality to another. The communities also follow the national government recommendation for the recovery of gathering spaces based on machizukuri to enhance citizen participation and community ties (Tatsuki and Hayashi 2000).

Aoki (2018) reviewed residents’ participation in the reconstruction of Onagawa City in the Tohoku area of Japan by measuring the level of participation based on Arnstein’s ladder of participation. Ishikawa (2015) described and reviewed the community-driven recovery process of Tamauranishi-Iwanuma City after GEJET-2011. Goto and Hiroi (2020) comparatively studied the recovery plans after the GEJET-2011 and Kumakoto Earthquake of 2016 and reviewed different considerations of community and gathering space recoveries in documents issued by municipal governments. These studies are good examples of community-driven recovery reviews; however, they were not comparative studies and did not focus on the importance of the construction of gathering spaces in implemented community recoveries.

## Hypothesis and methodology

**Fig. 3:**
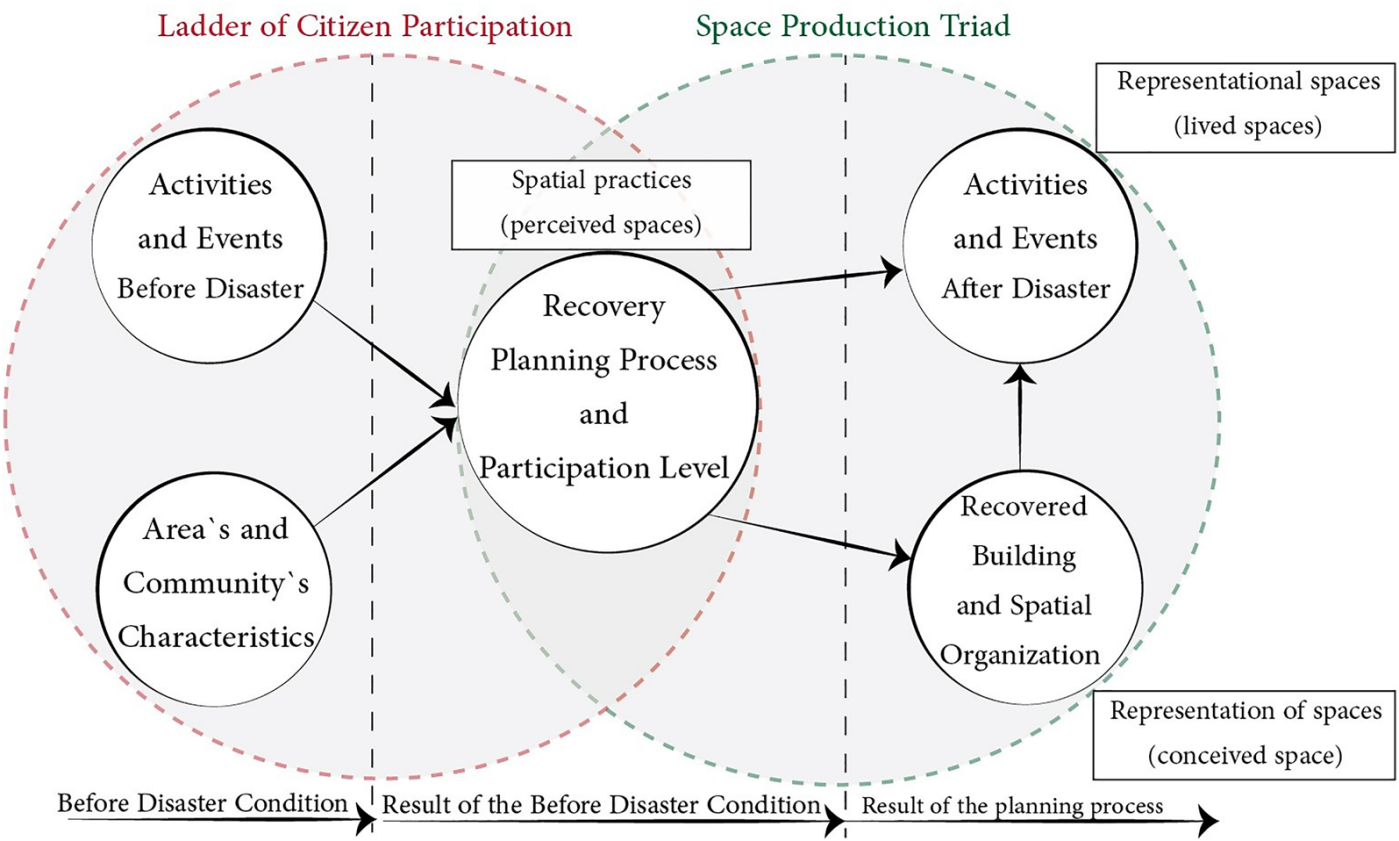
Hypothesis of the gathering space construction in cross-section with Ladder of Citizen Participation and Space Production Triad

The hypothesis identifies different recovery approaches and levels of participation of each embodied community and their outcomes of gathering space construction. The characteristics of activities, events, and areas before the disaster are assumed to impact the recovery planning approach and, accordingly, participation level during the recovery, activities, and events after a disaster. In other words, recovered gathering spaces and their spatial organizations result from post-disaster recovery planning and participation levels, which are affected by pre-disaster situations. At the latter impact, the recovered gathering spaces themselves can have an impact on activities and events after the disaster. Figure 3 shows this hypothesis and the impact of different aspects on the use of spaces based on the ladder of citizen participation and space production triad.

By extracting data collected through interviews and questionnaire surveys, this study attempts to identify different recovery scenarios and construction of gathering spaces based on two main background theories: Arnstein’s ladder of citizen participation and Henri Lefebvre’s production of space triad in selected case studies from the Tohoku region of Japan. The first part of the survey extracted the results of site visits and interviews. In this section, the first two factors affecting the constructed spaces are investigated and then categorized based on the recovery scenarios for spatial practices and the situation of gathering spaces for their representation. The second part demonstrates the results of the questionnaire surveys and extracts the factors of representational spaces for the case studies by gathering data from the respondents’ perspectives.

### Surveys

To understand the characteristics of the study area, we first collected timelines and events before and during the recovery process for each case study. This information was obtained based on documents issued by municipalities and scholars and through interview surveys conducted by the authors of this paper.

#### Sampling techniques and data collection

For the interview surveys, multiple locations were visited in March, May, July, and October 2019 and March 2020, and community leaders were interviewed at each visit. Semi-structured group interviews were conducted to gain more perspectives and a better understanding of the town, community, disaster experience, recovery scenarios, and gathering spaces. Each interview lasted for nearly two hours, and an attempt was made to collect the opinions and thoughts of all participants. The initial questionnaire was distributed in a trial questionnaire phase during these interviews, and the final questionnaire kit was designed based on the results of the trial phase. Questionnaire surveys were conducted in March and July 2020 and all households in the selected areas were covered. Respondents sent the kits back to the research laboratory through a pre-paid return envelope provided in the kits.

##### Analysis

To test this hypothesis, we first individually evaluated the case studies, aggregated the collected data, and analyzed the results by applying pattern matching for process and outcomes based on the hypothesis and discussions from the empirical literature and the use of logic models from the hypothesis model demonstrated in Fig. 3. Finally, we obtained cross-case synthesis methods for understanding the differences and similarities between multiple selected case studies (Yin 2017).

### Study area

For this study, four severely damaged areas were selected: Aoi-Higashimatsushima City, Tamauranishi-Iwanuma City, Machikata-Otsuchi City, and Shishiori-Kesennuma City in Miyagi and Iwate Prefectures in the Tohoku region.

**Fig. 4:**
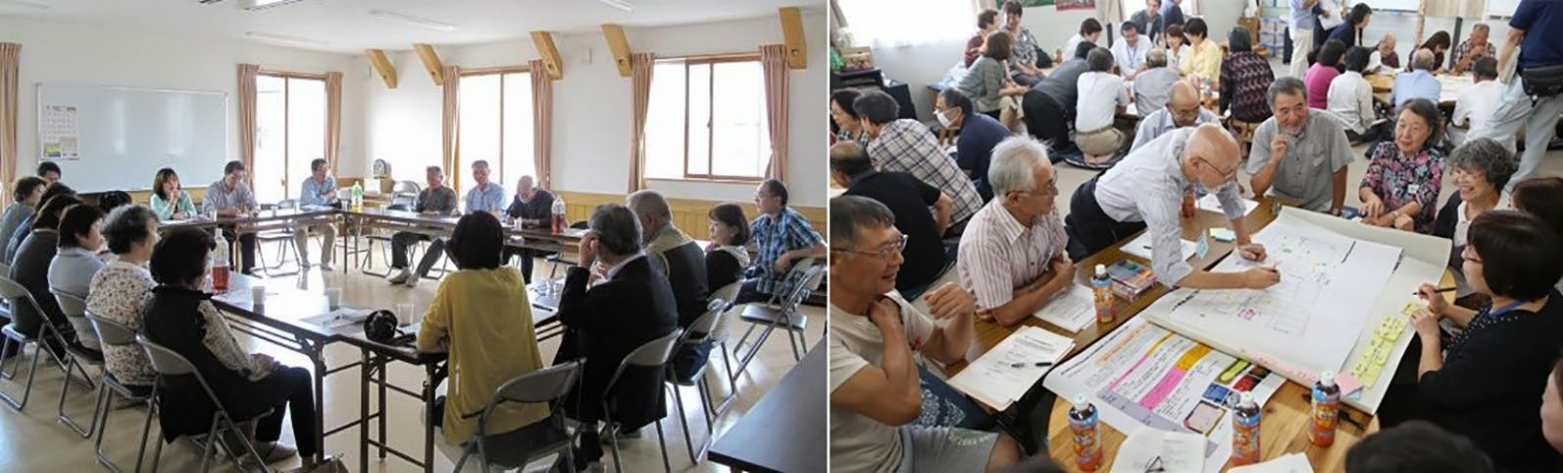
Collaboration of resident’s association and residents during recovery (left (JAEIC 2022), right (Mainichi 2022))

The Aoi district, located in Miyagi Prefecture, is a relocation site in the Omagari area, a coastal town in Higashimatsushima. Temporary housing was located at one site, and meeting places were provided by the neighborhood association for facilitating meetings and collect residents’ opinions regarding the neighborhood, gathering spaces, and recovery activities in the recovery plan. The recovery of Aoi was based on a community-driven approach, and residents and neighborhood associations actively participated in meetings. As a result, different types of gathering spaces were provided for the relocated areas for increasing the inclusivity, diversity, and accessibility of gathering spaces and activities offered to residents. The same diversity approach was adopted for housing recovery in this area; relocated households were placed in private detached, individual detached-style public, and apartment-style mass public housings. Figure 4 shows the images obtained through meetings during the recovery process.

**Fig. 5:**
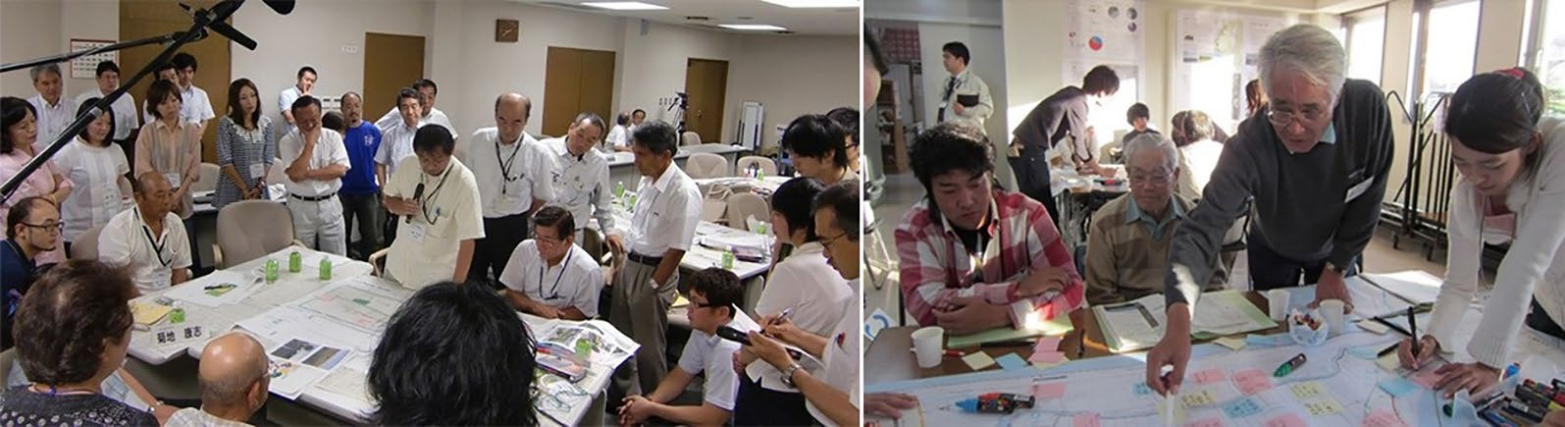
Collaboration of residents during recovery (left: Reconstruction 2022; right: Shokei 2022)

Tamauranishi District is a relocation site in the coastal area of Iwanuma City. Residents were moved to three temporary housing sites; however, community connections and meetings were retained for devising a community-driven recovery approach and reflecting on the residents’ opinions on the recovery plan. The recovered neighborhood maintained the proximity to the former area, albeit on a smaller scale. Three similar meeting places and parks offered inclusive and shared services within neighborhoods. Moreover, civic centers were shared with Tamauranishi and western Iwanuma for allowing public gatherings and events. Based on this community-driven recovery approach, the main housing type in this area (for both public and private housings) is individual detached-style buildings. Figure 5 shows the images obtained during the recovery process.

**Fig. 6:**
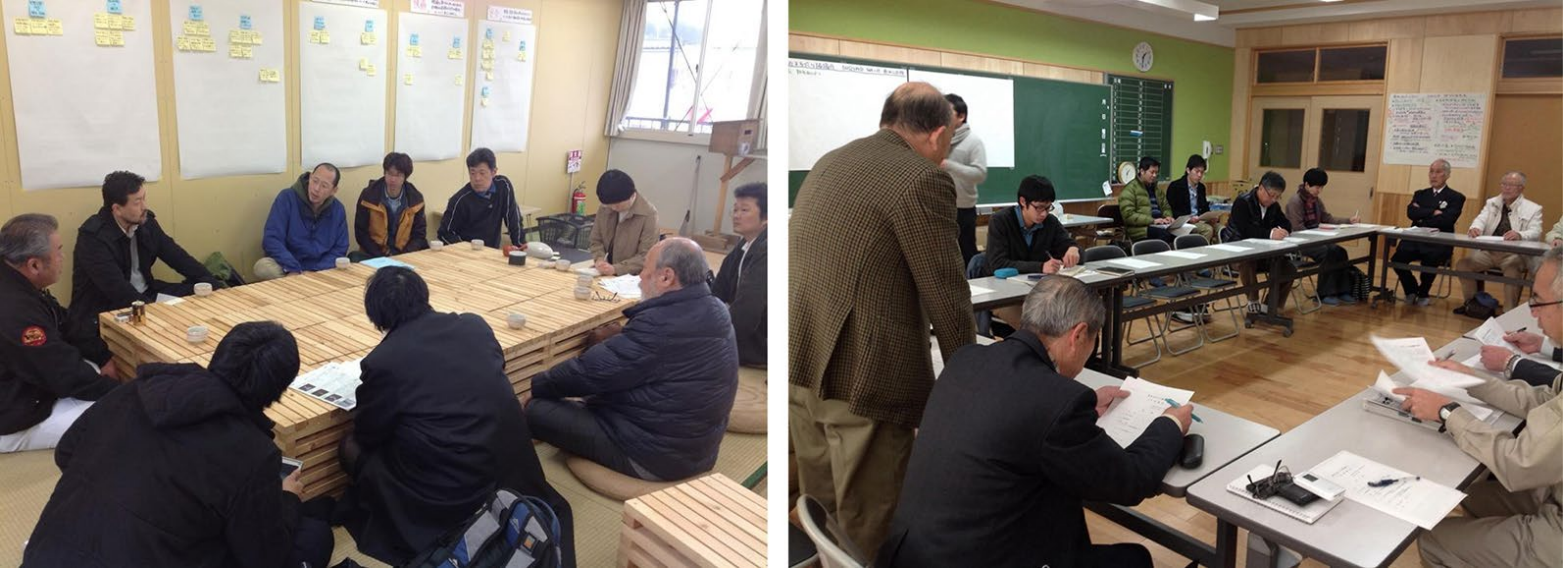
Meeting between Kobe city consultants and government officers (left) and first establishment of resident’s association in Shishiori area (right) (Kesennuma 2022))

The Shishiori neighborhood is located adjacent to the center of Kesennuma City in Miyagi Prefecture. The current public housing was constructed on temporary housing sites and former residential lots of those who did not move back after the disaster. The local government primarily administered the recovery plan, and residents had minor involvement in the process; however, the neighborhood association sought to hold inclusive meetings and activities in their building and at other sites for maintaining connections. The other gathering spaces provided in the area were civic centers and open spaces located at public housing sites. Figure 6 shows the images obtained during the recovery process.

The Machikata area is in the central part of Otsuchi Town, north of Iwate Prefecture, with a recovery plan mainly decided and managed by the local government. Two reinforced concrete apartment-style public housing and three other sites were in the northern part of a semi-detached and apartment style. A meeting place, accessible only by residents of the same public housing site, exists at each public housing site. “Oshacchi” complex is a community center/library places in a notable building. The complex faced Oshacchi Park and was planned and designed based on only three public workshops. The first workshop was held in the town office based on flyer advertisements and the Internet, the second was at a high school with students, and the third was in the main supermarket that remained undamaged by the disaster. The building serves as a public library, together with a community center of multiple rooms, kitchens, tatami rooms, music-dance rooms, and free Wi-Fi zones with maximum temporal accessibility. Rooms can be reserved at a low price for events and can be used from early morning until 10:00 pm. This building occasionally hosts local markets in parking lots for handicrafts, farmers, and food stalls.

### Questionnaire survey

Questionnaire surveys were conducted in March and July 2020. Ten years after the disaster, the questionnaire survey return ratio was predicted to be low. To address this issue, the distributions were decided not to be based on random selection but to cover all households regardless of disaster public and private housings in each recovered area. The questionnaire surveys were conducted during the initial lockdown of COVID-19 but were not significantly affected by the situation. The distribution and return ratios of the questionnaire surveys are listed in Table 1.

**Table 1:** Distribution and return ratio of questionnaire surveys

	Aoi-Higashi matsushima	Tamauranishi-Iwanuma	Kesennuma Shishiori	Otsuchi Machikata
*Disaster public housing*				
Distributed	307	145	246	359
Returned	44	39	44	49
*Other type*				
Distributed	235	209	270	297
Returned	53	64	34	35
*Total*				
Distributed	542	354	516	656
Returned	97	103	78	84
Return (%)	18	29	15	13

In total 2076 questionnaires were distributed, and 362 (18%) were returned. The results of the questionnaire showed that most respondents were over 65 years old, and the gender of the respondents showed equal distribution among all cases. The results of respondents’ dwelling situation showed that the number of respondents who lived in private dwellings in Tamauranishi-Iwanuma and Aoi-Higashimatsushima was higher than in the other cases. The demographic results of the questionnaire distribution are presented in Table 2.

**Table 2:** Summary of demographic results of the questionnaire survey results

		City				Total
		Higashimatsushima-Aoi	Iwanuma-Tamauranishi	Kesennuma-Shishiori	Otsuchi-Machikata	
*Age ratio*						
Under 65	*N*	48	35	29	35	147
	(%)	50	35	37	43	42
Over 65	*N*	48	64	49	46	207
	(%)	50	65	63	57	59
Total	*N*	96	99	78	81	354
*Gender*						
Male	*N*	49	58	43	41	191
	(%)	51	57	55	49	53
Female	*N*	47	43	35	42	167
	(%)	49	43	45	51	47
Total	*N*	96	101	78	83	358
*Dwelling*						
Private housing	*N*	53	64	34	35	186
	(%)	55	62	44	42	51
Public housing	*N*	44	39	44	49	176
	(%)	45	38	56	58	49
Total	*N*	97	103	78	84	362

### Results and findings

After reviewing the cases and aggregating the collected data, we examined the analysis results and represented them through the two main background theories of this study, Arnstein’s ladder of citizen participation and Lefebvre’s production of space.

#### Spatial practices (perceived spaces) force powered by level of participation

After summarizing the cases, we used Arnstein’s ladder of participation as an indicator for identifying the levels and categories of residents’ participation in the spatial practices of gathering spaces and their initiative for recovery scenarios, as explained in Fig. 2. Based on these measurements and observations, the case studies were divided into two groups: community-driven and government-led recovery scenarios. Case studies with higher levels of participation at the citizen level were considered community-driven, whereas case studies with lower levels were considered government-led projects.

All cases have at least a consultation level of participation in their recovery plans, which is common among global cases. Aoi-Higashimatsushima exhibited the highest level of participation, because this area provides citizens with empowering and decision-making controls. Tamauranishi-Iwanuma, reflecting the partnership of citizens in the recovery plan, is in the second stage. Shishiori-Kesennuma and Machikata-Otsuchi, being at the level of consultation and informing residents, were at the lowest level among all cases. The reconstruction and recovery details for each case study are summarized in Table 3. Each case study is examined based on the aforementioned factors and the results of the questionnaire surveys in the next section.

**Table 3:** Summary of the recovery initiatives of the case studies- spatial practices

Area	Reconstruction type	Participation process	LOP^a^	Recovery scenario
Aoi-Higashimatushima	Relocation, compact city, no business provided	Constant meeting with residents, decisions made and executed by residents, before disaster preparation. Gathering spaces opened on 2015 after completion of housings	8	Community-driven
Tamauranishi-Iwanuma	Relocation, compact city, no business provided	Constant meeting with residents, decisions based on residents` opinion and partnership. Gathering spaces opened on 2015 after completion of housings	6	Community-driven
Shishiori-Kesennuma	Land readjustment, relocation, small retails provided	Decisions based on government’s perspective. Consultation and informing with the residents for some details. Gathering spaces opened on 2017 after completion of housings	4	Government-Led
Machikata-Otsuchi	Land readjustment, relocation, small retail provided	Decisions based on government’s perspective. Consultation and informing with the residents for some details. Gathering spaces opened on 2018 after completion of housings	4	Government-Led

^a^Level Of Participation: Based on participation based on Arnstein’s ladder of participation non-Participation ((1) Manipulation and (2) Therapy), Tokenism ((3) Informing, (4) Consultation and (5) Placation), Citizen Power ((6) Partnership, (7) Delegated Power and (8) Citizen Control)

**Fig. 7:**
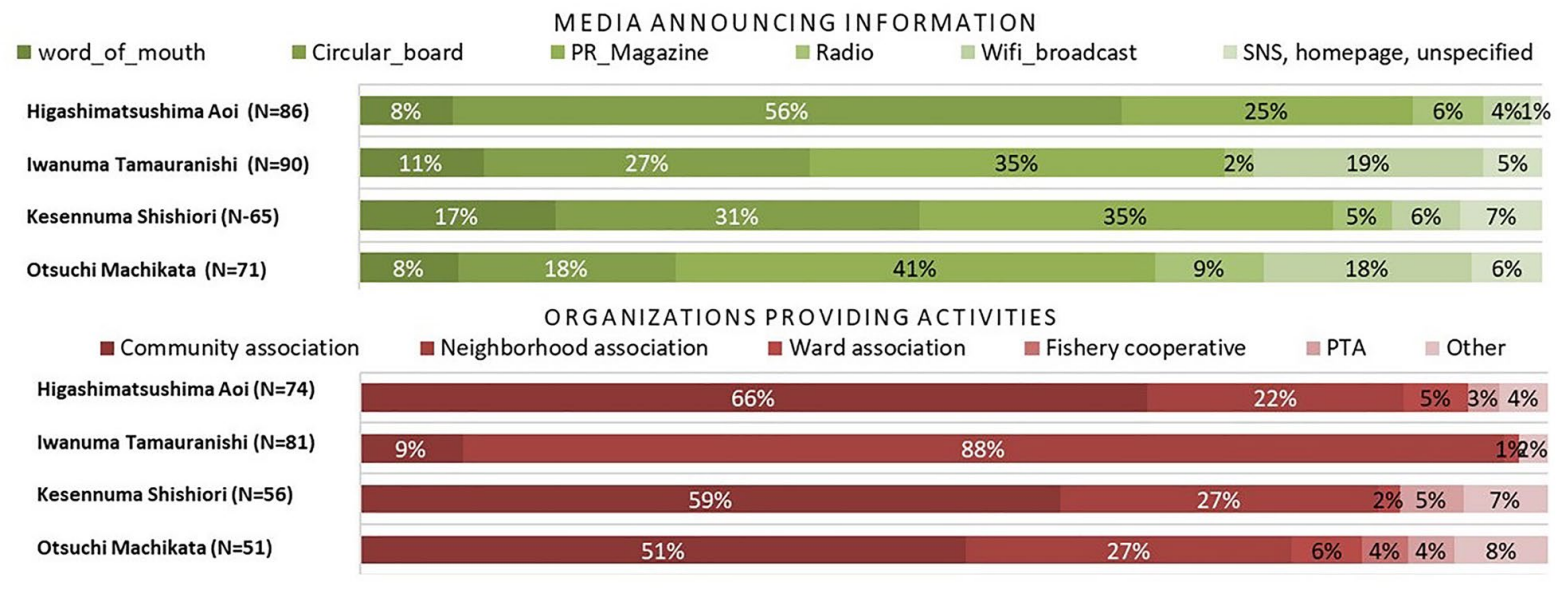
Type of media announcing activities and organization that carried out the gathering activities

To identify the accessibility of information regarding events and functions of gathering spaces, we questioned the residents about the media mean that was used by initiatives to announce services and activities and the organizations that oversaw gathering activities in gathering spaces (also in Fig. 7). In all the areas, circular boards and PR magazines were the main means of media for announcing the services, and in Tamauranishi-Iwanuma and Otsuchi machikata Wi-Fi broadcasts were selected. In community-driven cases, the selection of neighborhood associations was the highest, and in government-led cases, the selection of community associations was the highest. The selection of PTA (Parent-Teacher association) was higher in government-led cases.

#### Representation of spaces (conceived space) embodied in gathering spaces

**Fig. 8:**
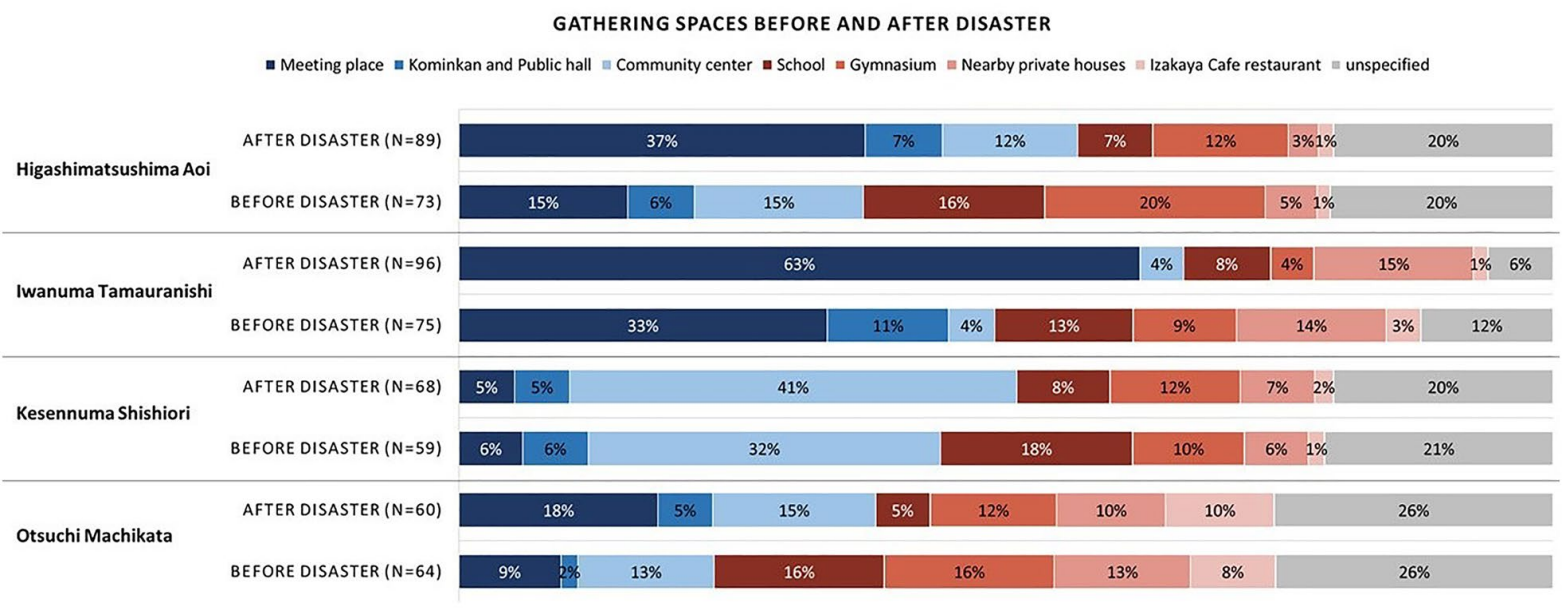
Different gathering spaces that respondents go to before and after disaster, multiple choice

Because the recovery of gathering spaces is the main spatial purpose of this study, the questionnaire survey included questions about the types of gathering spaces before and after the disaster. Figure 8 shows the results of gathering space choices by respondents before and after the disaster based on multiple-choice questions. The results showed that the selection of schools and gymnasiums decreased after the disaster in all the areas. In addition, selection of meeting places (small gathering spaces) increased in community-driven cases (Aoi-Higashimatsushima, Tamauranishi-Iwanuma), while selection of community centers increased in other cases.

In addition, to collect the spatial configuration, diversity, and allocation of gathering spaces, the details of the gathering spaces of each case study were documented by the authors during field visits. The observations showed that community-driven cases provided evenly allocated gathering spaces and separated Hiroba (a wide-open area) with equal accessibility to different users in the surrounding housing blocks. In these cases, gathering spaces are in a high administrative connection with others. Conversely, in government-led cases, even though several gathering spaces are provided, the main gathering space is centralized and not well connected with other gathering spaces. Furthermore, the main gathering space provides large-scale Hiroba (wide-open areas) along with gathering spaces and services to outside communities. A diagrammatic representation of accessibility and gathering space allocation for each case study is summarized in Table 4.

**Table 4:**
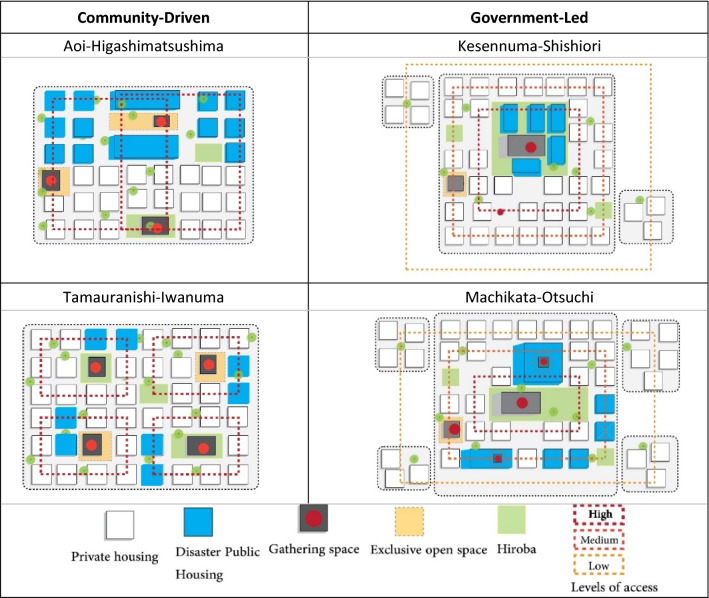
Summary of production of gathering spaces in case studies

Next, we mapped the micro-spatial dimensions of each gathering space and Hiroba (wide-open area) and presented them based on modular dimensions and in comparison with one another. The details of the spatial configuration and diversity of the gathering spaces are listed in Table 5. In community-driven cases, the number of gathering spaces is higher than in government-led cases, and spaces are provided for different functions, especially for planned activities. In most cases, these provide exclusive open spaces and limited Hiroba (wide-open area), but the configuration of such spaces is mostly at the pocket-size level. In government-led cases, a few small gathering spaces and a large main gathering space with diversified spaces for planned activities and spontaneous use of space, such as lounges and Hiroba (wide-open spaces), exist.

**Table 5:**
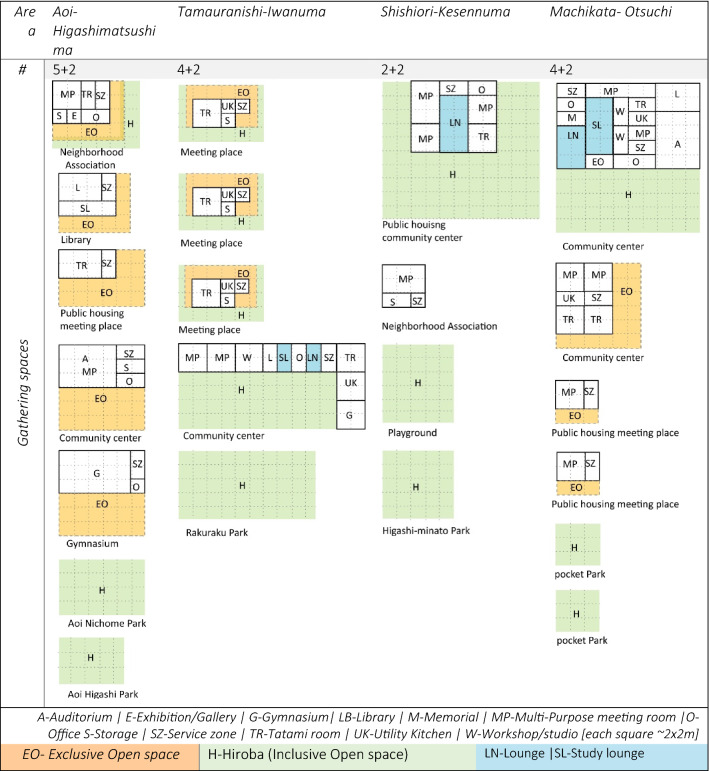
Representation of gathering spaces in each case study of spaces based on observations and field visits

#### Representational spaces (lived spaces) embodied as individual’s experience

**Fig. 9:**
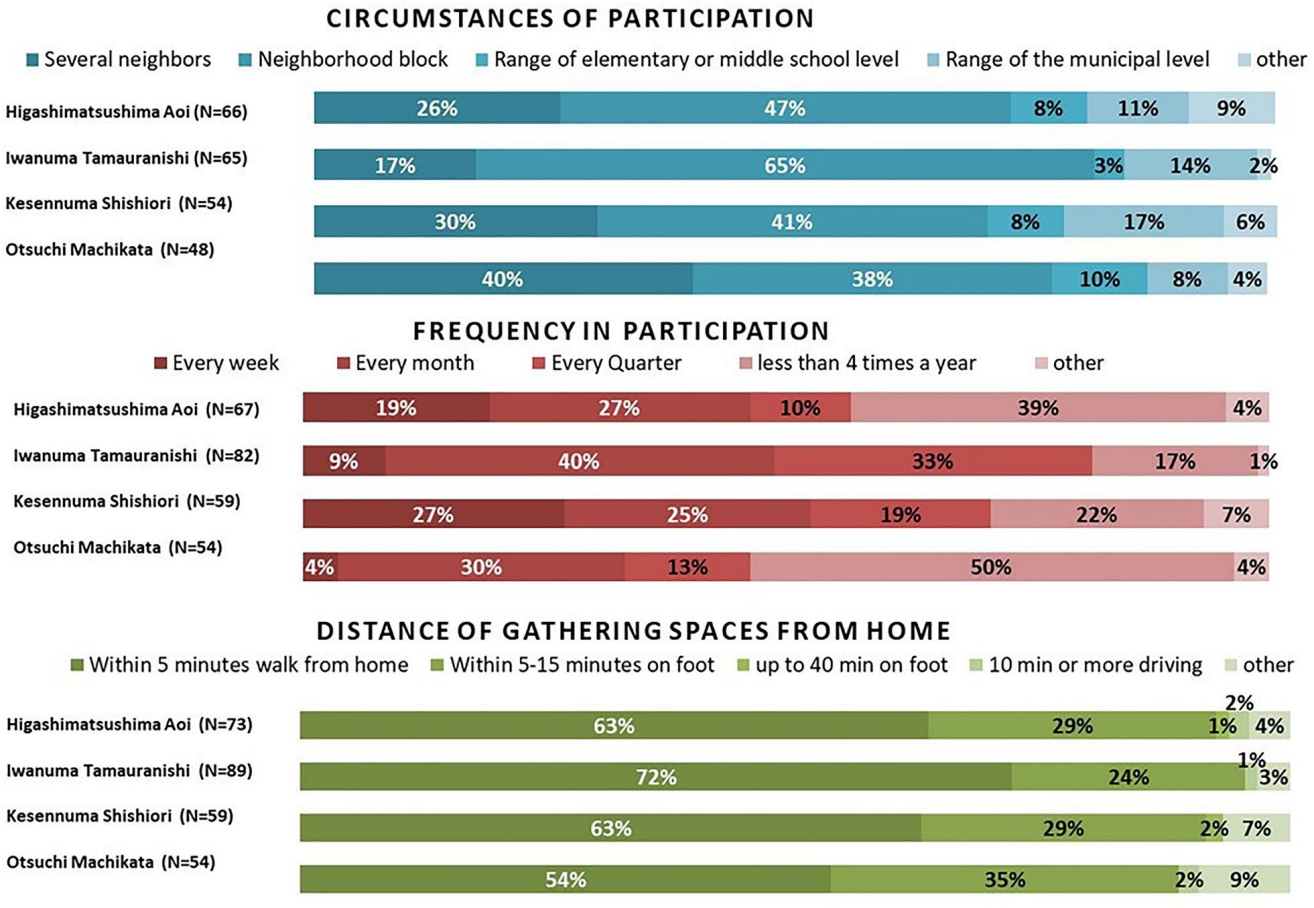
Range of activity circumstances, frequency in participation, and distances of gathering spaces from homes

The final part of the triad of space production emphasizes functions of space for users. In this regard, questionnaires included questions regarding residents’ experiences with gathering spaces and activities they participated in before and after the disaster. Figure 9 shows the circumstances of the gathering activities, frequency of participation, and distances of gathering spaces from homes. In community-driven cases, the neighborhood block has the highest number, whereas in government-led cases, a similar distribution exists between neighborhood blocks and several neighbors. These cases also selected a range of middle school districts that were more than community-driven cases. Regarding the frequency of participation in gathering activities, with a similar distribution among cases, community-driven cases participated more than government-led ones. While a very similar distribution existed among both categories, community-driven respondents lived closer to gathering spaces.

**Fig. 10:**
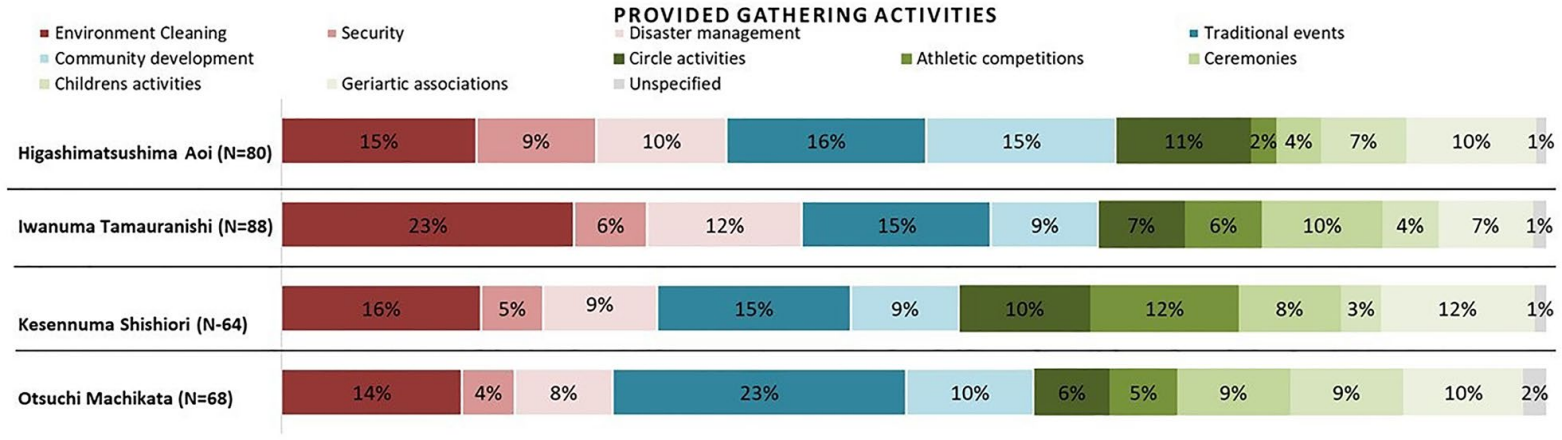
Provided gathering activities by organizations, multiple choice

Figure 10 shows the selection of gathering activities provided by organizations. The results show that the main gathering activities provided by organizations were environmental cleaning and traditional events. The results show that while respondents chose similar gathering activities before and after the disaster, the selection of community development activities increased after the disaster. In community-driven cases, the provision of security and circular activities increased after the disaster.

**Fig. 11:**
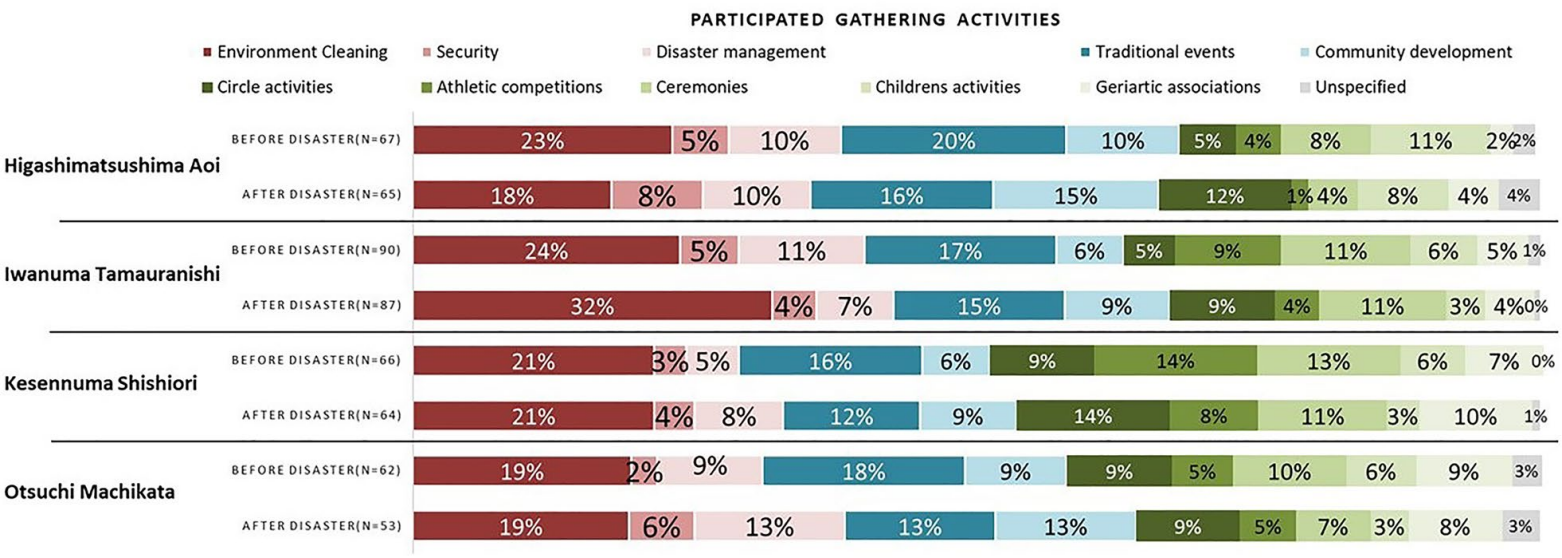
Gathering activities chosen to participate, multiple choice

Figure 11 shows the gathering activities selected for participation by respondents. The selection of traditional events decreased after the disaster in all areas, and environmental cleaning activities were chosen as the main activities for participation. In addition, the selection of community development and circular activities increased after the disaster.

By individual pattern matching of cases and cross-case analysis, we identified similar patterns in case studies that initiated similar community-driven and government-led recovery scenarios. The cases of Aoi-Higashimatsushima and Tamauranishi-Iwanuma adopted a community-driven approach, while Shishiori-Kesennuma and Machikata-Otsuchi adopted a government-led approach for their recovery plans (Table 3).

Our results indicate that in community-driven cases, the selection of neighborhood associations is higher, and in government-led cases, the selection of community associations is the highest. The selection of PTA was higher in government-led cases. In community-driven cases, the neighborhood block has the highest number, whereas in government-led cases, a similar distribution exists between neighborhood blocks and several neighbors. These cases also selected a range of middle school districts that were more than community-driven cases.

In both categories, gathering spaces are combined with a main large-scale gathering space that provides more gathering services for the community and smaller gathering spaces, but the connections between and access to different gathering spaces are different (Fig. 9; Tables 4, 5).

In the cases of community-driven recovery initiatives, several gathering spaces are evenly allocated in housing blocks containing exclusive open spaces and extended to pocket size Hiroba. Furthermore, they had constant connections with the main gathering space. The territory providing services for community-driven cases is mostly within the boundaries of the community and for their own community members. In government-led cases, the main gathering space is the most active and accessible gathering space, provides large public open spaces in connection with the building, and provides services to visitors from outside the community. In contrast, smaller gathering spaces in government-led cases are not as active and accessible as the main gathering space and are not well managed and actively connected with one another (Fig. 9; Tables 4, 5).

In terms of representational spaces, respondents from different communities selected similar gathering activities before and after the disaster but different activities to participate in from one another. The selection of traditional events decreased after the disaster in all areas, and environmental cleaning activities were the main activities for participation. In addition, the selection of community development and circular activities increased after the disaster. In government-led cases, the main gathering spaces provided spaces for passive participation, free of choice activities, and planned active participation spaces, such as lounges and Hiroba (inclusive open space), which affected the experience of the respondents in using the gathering spaces.

## Discussion

Since the questionnaire surveys were conducted almost ten years after the disaster, the return ratio of the effective questionnaires was 18%. This ratio might have been impacted by the time elapsed after the disaster, a lower level of interest in participating the surveys, and the early restrictions of COVID-19 countermeasures in the selected communities. The reconstruction of the selected communities and gathering spaces over ten years may have affected the results and performance of the gathering spaces because community activities may have changed during this period.. In some areas, depending on the recovery scenario and reconstruction method, the completion and introduction of gathering facilities took longer than in other areas.

### Importance of gathering spaces

Japanese national and local governments (Tatsuki and Hayashi 2000) have encouraged the recovery of gathering spaces in post-disaster planning. This is a notable effort that reaffirms the views of global scholar on the importance of public and gathering spaces (Holub 1991) and the role they play in helping disaster affected areas to function as a community transitioning from disaster emergency to recovery (Haas et al. 1997).

By reviewing the process and scenario of recovery planning in each category of case studies (Table 3), we understand that in community-driven cases, where continuous meetings were held among residents and stakeholders, a post-disaster sense of community was established, resulting in shared values among members (Klinenberg 2018). Throughout these community-driven processes, residents are in charge of their own place and activity planning; they interact and discuss with other community members about their needs, and present individual perspectives in multi-functional gathering spaces (Low and Altman 1992).

In the studied areas, we found that issues, such as displacement of residents or lack of accessibility, lack of community recovery, and lack of emotional support (Nelan and Schumann Iii 2018), were not mentioned by respondents from community-driven cases; however, in government-led cases, displacement and inaccessibility could be identified.

### Production of gathering space

We reviewed the theory of space construction through study areas and identified residents’ participation level as a contributing factor for spatial practices in the space production triad (Fuchs 2019) and a catalyzer for the co-production and co-design of shared spaces (O'Reilly et al. 2022; Roy and Ong 2011). We divided case studies into two categories: community-driven (fully participatory, co-productive) and government-led (top-down, non-participatory) (see Fig. 2). However, we found that even in government-led cases, stakeholders held consultation meetings with the residents and informed the participants regarding their plans (Table 3). This could be seen as the main purpose of participatory town planning, which is encouraged by the national government and not limited only to case studies in this research and the particular disaster it focuses on (Aoki 2018; Goto and Hiroi 2020; Ishikawa 2015; Maly 2018; Murakami et al. 2014; Tatsuki and Hayashi 2000).

Theanalysis of representation of spaces in the studied areas shows that cases with similar recovery scenarios (community-driven and government-led) had similar allocations and configurations of gathering spaces. In community-driven cases, gathering spaces are smaller in size, are allocated for events in the community districts, and are placed at a considerable distance from the residents’ houses (Table 4). However, in government-led case studies, the main gathering spaces are centralized, larger in size, and provide different levels of access to users at varying distances from them (Table 4). These results are supported by the questionnaire survey results in which respondents living in community-driven cases go to smaller gathering spaces and within smaller boundaries, participate more often in gathering activities (Figs. 8, 9), and select more diverse types of activities in general, compared to government-led cases (Figs. 10, 11). Indeed, these results support the suggestion of global scholars on how high levels of residents’ participation and co-production methods of space recovery ensure inclusivity and accessibility, consideration of the needs of majority and minority members of a community in spatial planning and utilization, and exploration of diverse capacities that a community can reach by engaging in the planning (Fung 2015; Gururani and Kennedy 2021; Murphy et al. 2022; O'Reilly et al. 2022; Reed et al. 2022; Roy and Ong 2011).

In addition to these results, and those discussed in the literature, inclusive open spaces or Hiroba (wide-open space) are understood differently in the Japanese context (Aota 2012; Dimmer 2012; Kuma et al. 2015; Okabe 2017); however, we could observe a paradigm shift in the studied cases. In community-driven gathering spaces, open spaces are exclusive to the allocated gathering space and follow the curfew of the respective space, and Hiroba (a wide-open area) is provided as pocket parks. On the other hand, in government-led gathering spaces, the centralized gathering space is accompanied by a large inclusive open space or Hiroba (wide-open space) that welcomes users, regardless of the limitations of the respective gathering space (Table 4). In fact, these government-led Hirobas (wide-open spaces) are very close to the global definition of public spaces and function as such spaces (Aota 2012; Holub 1991).

By observing the microspaces in the studied areas, the community-driven gathering spaces were found to have clearly designed interior spaces for planned activities and active and purposeful participation of users was required in these spaces. In government-led gathering spaces, interior spaces exist for passive participation and spontaneous gatherings of users (Table 5). These are important paradigm shifts in the Japanese context, where the definition and the process of realization of public spaces are different from the global context. This indicates that because governments control the access to resources and infrastructure, they should contribute by providing more flexible uses of public spaces to the general public.

## Conclusion

This paper investigated the production of gathering spaces after GEJET-2011 in selected areas and explored the connections between the triad of space production and residents’ participation in the implementation of spaces. The results of the surveys regarding the triad of space in the case of gathering spaces demonstrate that the spatial practices (perceived spaces) in the studied areas are community-driven and government-led recovery scenarios; the representation of spaces (conceived space) is the constructed space, such as the number and configuration of gathering spaces, interior rooms, and Hiroba (wide-open areas), and the representational spaces (lived spaces) are the experiences of residents in the way they use those spaces and engage in gathering activities. Scholars emphasize that gathering and public spaces are important for strengthening community ties, helping enhance residents’ participation, and establishing a sense of community, place attachment, and representation (Low and Altman 1992). In the studied areas, case studies based on functional gathering spaces are among the good examples of global cases (in comparison with no gathering space at all). In Japan, with the benefit of the experience of recovering after the 1995 Hanshin-Awaji earthquake, it was possible to avoid the negative impacts of lacking gathering spaces and community contacts (Murakami et al. 2014; Tatsuki and Hayashi 2000).

According to the introduced hypothesis and Fig. 3, the process of space production and recovery could impact the realization of spatial environments and their utilization for different shared functions by the users. These case studies are good examples of the contribution of the process of gathering space construction to greater benefits for users. First, the characteristics of the community and area before the disaster, together with the level of gathering activities and events, can lead to the planning and administration process after the disaster and determine the participation level of the residents and spatial practices. These planning and participation results can contribute to the building and spatial configuration of gathering spaces and the upcoming activities and events provided to the residents.

Second, the representation of spaces (conceived space) could be affected by spatial practices (community-driven, government-led), resulting in different buildings in the recovered communities. The representation of spaces in community-led cases follows a semi-equal allocation of gathering spaces with pocket-size open spaces, whereas in government-led cases, centralized gathering spaces surrounded by large open spaces (Hiroba) exist.

Third, the representational space (lived spaces) could be achieved by varying the process; for example, residents from community-driven cases receive equal access and spatial configuration and diversified gathering spaces and participate more in gathering activities; residents of government-led cases have different levels of access to the main centralized gathering space and less frequently participate in the activities. Furthermore, in government-led gathering spaces, residents benefit from spaces aiming at accepting passive participation, such as lounges and largescale Hiroba.

These findings are important to researchers and planners in the field of space design and governance development, and could contribute to the utilization of better public and gathering spaces in the future. This study concludes that the process of gathering space construction after GEJET-2011 may have an impact on the long- and short-term experiences of the affected communities in planning participation, accessing gathering spaces, and benefiting from different spatial configurations and diversification.

The findings of this study suggest that stakeholders and researchers should consider collaborations between residents’ associations and a community-driven approach to achieve better community recovery through high-quality gathering spaces. In addition, based on this research, community-driven gathering spaces are recovered closer to the residents’ opinions and therefore may address the mid- and long-term needs of community members in terms of proximity, accessibility, and activity diversity more thoroughly than government-led cases with central authorization and administrative complexities for the community and the surroundings towns.

However, the debate on whether an adequate participatory and co-productive process of public space provision or government-provided large-scale public space with optional spaces could result in significant benefits for users in the long term is ongoing.

## Data Availability

The initial data is available in the published report by Hokugo and others (24) from a prior questionnaire survey covering different aspects of post-GEJET2011 long-term disaster recovery aspects.
